# Effects of feedstock and co-culture of *Lactobacillus fermentum* and wild *Saccharomyces cerevisiae* strain during fuel ethanol fermentation by the industrial yeast strain PE-2

**DOI:** 10.1186/s13568-018-0556-9

**Published:** 2018-02-16

**Authors:** Vanda R. Reis, Ana Paula G. Bassi, Bianca C. Cerri, Amanda R. Almeida, Isis G. B. Carvalho, Reinaldo G. Bastos, Sandra R. Ceccato-Antonini

**Affiliations:** Dept Tecnologia Agroindustrial e Socio-Economia Rural, Universidade Federal de São Carlos-Centro de Ciencias Agrarias, Via Anhanguera, km 174, Araras, SP 13600-970 Brazil

**Keywords:** Ethanol, Fermentation, Wild yeasts, Bacterium, Feedstock, Sugars

## Abstract

Even though contamination by bacteria and wild yeasts are frequently observed during fuel ethanol fermentation, our knowledge regarding the effects of both contaminants together is very limited, especially considering that the must composition can vary from exclusively sugarcane juice to a mixture of molasses and juice, affecting the microbial development. Here we studied the effects of the feedstock (sugarcane juice and molasses) and the co-culture of *Lactobacillus fermentum* and a wild *Saccharomyces cerevisiae* strain (rough colony and pseudohyphae) in single and multiple-batch fermentation trials with an industrial strain of *S. cerevisiae* (PE-2) as starter yeast. The results indicate that in multiple-cycle batch system, the feedstock had a minor impact on the fermentation than in single-cycle batch system, however the rough yeast contamination was more harmful than the bacterial contamination in multiple-cycle batch fermentation. The inoculation of both contaminants did not potentiate the detrimental effect in any substrate. The residual sugar concentration in the fermented broth had a higher concentration of fructose than glucose for all fermentations, but in the presence of the rough yeast, the discrepancy between fructose and glucose concentrations were markedly higher, especially in molasses. The biggest problem associated with incomplete fermentation seemed to be the lower consumption rate of sugar and the reduced fructose preference of the rough yeast rather than the lower invertase activity. Lower ethanol production, acetate production and higher residual sugar concentration are characteristics strongly associated with the rough yeast strain and they were not potentiated with the inoculation of *L. fermentum*.

## Introduction

The Brazilian industrial process for fuel ethanol production has certain peculiarities such as the maintenance of non-aseptic conditions and cell recycling between fermentative cycles. These characteristics are known to contribute significantly towards the development of contamination by wild-type yeasts and bacteria. Although a wide variety of yeasts are found to be present in the fermentation medium, only a few are able to compete with the selected strains of *Saccharomyces cerevisiae* that are known to dominate and persist in the fermentative environment. Amongst non-*Saccharomyces* yeasts that are known to contaminate industrial processes, the species *Dekkera bruxellensis* has received increased attention for its outstanding growth capability and poor fermentative ability under low-oxygen conditions (Souza-Liberal et al. 2007; Pereira et al. 2012; Meneghin et al. 2013).

A particular biotype of *S. cerevisiae* that is frequently found in ethanolic fermentation processes displays cells arranged in clusters and forms rough colonies on solid medium. Reduced fermentation rates and high residual sugar content are associated with the presence of these contaminating yeast strains in the fermentation medium (Reis et al. 2013). In spite of being *S. cerevisiae* strains, these yeasts are classified as contaminants because of the operational problems that arise due to their specific characteristics such as formation of scum, cell flotation or sedimentation, and reduced efficiency of cell separation (using centrifugation) from the fermentation medium. Yeasts with such features—rough colonies and pseudohyphal growth—are capable of dominating the fermentation process and replacing the starter yeast strain (Basso et al. 2008). These yeasts display a high degree of cell sedimentation that results in problems that are similar to those observed with flocculent yeasts. On a cautionary note, cell aggregation that originates from pseudohyphae should not be confused with those that results from flocculation. The formation of cell chains results from the failure of the young bud to separate from the mother cell (Soares 2010) which leads to the daughter cell remaining attached to the parent after mitosis. These yeasts are also referred to by the term ‘snowflake yeasts’. As cell chains continue to elongate with each subsequent cell division, the tension between the cells rises till the tension force exceeds the cell–cell adhesion resulting in the release of multicellular propagules (Ratcliff et al. 2015).

In addition to yeasts a wide variety of contaminating bacteria have also been found during the fermentation processes. These bacteria are usually Gram-positive rods and acid-producers that display robust growth profiles at pH values in the vicinity of 5.0, which, incidentally, is the pH of the fermentation medium (Andrietta et al. 2011). It is well established that a majority of the bacterial contaminants belongs to the genus *Lactobacillus* (Lopes et al. 2016). This genus, depending upon the pathway utilised for sugar metabolism, can be split into two major metabolic categories: (a) heterofermentative bacteria, and (b) homofermentative bacteria (Kandler 1983; Costa et al. 2008; Lucena et al. 2010). In comparison to the homofermentative type of bacteria (*Lactobacillus plantarum*), heterofermentative *L. fermentum* has a more deleterious impact on ethanolic fermentations that employ cell recycling (Basso et al. 2014). In an industrial process, *L. fermentum* was responsible for both cell co-aggregation as well as for the high levels of organic acids (Carvalho-Netto et al. 2015). There is an effect of the number of cells to result in industrial decrease of ethanol yield. Amorim et al. (2009) estimated that 20,000 litres of ethanol are lost per day in a medium-sized Brazilian distillery when bacterial contamination increases from 10^7^ to 10^8^ cells mL^−1^.

With respect to the industrial environment, it is well established that in fermentors both bacteria and wild yeasts co-exist with the starter yeast especially selected for the fermentation process. A significant amount of data exists with regard to the roles assayed by bacteria or wild yeasts alone but the information available on if and how they interact with each other and also with the starter yeast is scanty and has not received the special attention that is warranted. A few published reports that analyse the co-occurrence of *Lactobacillus* and *D. bruxellensis* in fermentation processes and evaluate its implications on the ethanol yield can be found in literature (Passoth et al. 2007; Souza et al. 2012; Tiukova et al. 2014). The multitude of interactions possible among bacteria, wild and starter strains of *S. cerevisiae* remains to be satisfactorily elucidated.

The composition of the feedstock may be a factor that interferes with microbial interactions during the fermentation process. The feedstock is comprised of either sugarcane juice or molasses, or more commonly, a combination of both substrates (Andrietta et al. 2011). In Brazilian ethanol-operating units, the fermentation substrate is not standardised to meet the needs of the starter yeast and its usage is dependent upon the balance between ethanol and sugar production processes. Sugarcane juice, obtained from the pressing of sugarcane stalks, presents a physico-chemical composition that varies according to the harvest period, stalk sanity, extraction method, microbial contamination, cane varieties, etc. (Martini et al. 2010, 2011; Andrietta et al. 2011). Molasses, a by-product of the sugar industry, has higher nutrient content but it is known to contain several secondary products such as organic acids, hydroxymethylfurfural, melanoidines, etc., all of which may interfere with the fermentative process (Tosetto 2008; Andrietta et al. 2011). The presence of a particular compound in the feedstock content has the potential to exert a selection force in the fermentors that may favour some specific microorganisms (Tosetto 2008).

In the context of microbial contamination and fermentation feedstock, our study was conducted with the aim of verifying the impact of co-inoculation of *L. fermentum* and a wild strain of *S. cerevisiae* on fermentation trials conducted by PE-2 starter yeast strain. It was also our objective to verify if the substrate, sugarcane juice or molasses, interferes with the interactions amongst the microorganisms. Fermentations with and without cell recycle were also evaluated. Speculations regarding the possible causes for incomplete fermentations are also presented in the text.

## Materials and methods

### Microorganisms

Two strains of *S. cerevisiae* were utilised during the course of these experiments: (a) the industrial yeast PE-2 (kindly provided by Dr Antonio Joaquim Oliveira from Fermentec S/A, Piracicaba—SP—Brazil) and, (b) a rough-colony yeast strain, isolated from a fuel ethanol facility in São Paulo State, Brazil. The latter (originally termed ‘strain 52') was kindly provided by Dr. Ana Teresa Burlamaqui Faraco Antonangelo (BPI Biotecnologia, Botucatu—SP—Brazil), identified by the sequencing of the D1/D2 region of the large subunit (26S) rRNA by Dr Fernando Carlos Pagnocca (Universidade Estadual Paulista, Rio Claro—SP—Brazil), and deposited at the culture collection ‘Coleção de Culturas Tropical’ of ‘Fundação André Tosello’, Campinas—SP—Brazil (CCT7787). The GenBank access number for this nucleotide sequence is KY315817. Additionally, a strain of *L. fermentum* CCT0559 (ATCC9338) was also used. The yeast and bacterium strains were maintained on YPD (10 g L^−1^ yeast extract, 20 g L^−1^ glucose, 20 g L^−1^ peptone, 20 g L^−1^ agar) and MRS (Man-Rogosa-Sharpe) Medium (Himedia^®^) slants at 4 °C, respectively. Both bacterial as well as yeast strains were continuously transferred to new growth medium before the experiments.

### Fermentative tests in a single-cycle batch system

Yeast inoculum was prepared by separately inoculating two loops of each yeast strain in 125-mL flasks containing 50 mL of the multiplication medium (clarified sugar cane juice or molasses with approximately 4 g 100 mL^−1^ of total reducing sugars, pH 5.5, as supplied by a local fuel ethanol-producing unit, and sterilized at 120 °C for 20 min). The inocula were incubated at 30 °C, 160 rpm for 24 h. The yeast cell mass was separated from the liquid growth medium by centrifugation at 580*g* for 5 min. This cell mass was again incubated under the conditions described above. The flasks were maintained at 30 °C, 160 rpm. The growth medium was continuously renewed until the amount of 10 g L^−1^ wet mass was achieved to be added to the medium. The wet mass was resuspended in the fermentation medium (clarified sugar cane juice or molasses with approximately 14–17 g 100 mL^−1^ of total reducing sugars, pH 4.5, as supplied by a local fuel ethanol-producing unit, and sterilized at 120 °C for 20 min) and added to 500-mL flasks containing 200 mL of medium, in semi-aerobiosis conditions (cotton-capped flasks). The initial cell concentration was computed to be approximately 10^8^ CFU mL^−1^ for the starter yeast and 10^6^ CFU mL^−1^ for the rough yeast.

The preparation of *L. fermentum* inoculum involved the execution of two steps as elaborated below: the bacterium was grown in MRS medium and incubated at 35 °C, 160 rpm for 24 h. The bacterial mass was then transferred to the multiplication medium (sugarcane juice or molasses) and incubated at 35 °C, with agitation, till an optical density (540 nm) of about 0.5 was achieved. The cell suspension was centrifuged at 1160*g* for 10 min (at 4 °C) and the mass was resuspended in the fermentation medium. The initial cell concentration was about 10^6^ CFU mL^−1^.

The following fermentation trials were carried out: (a) with PE-2 exclusively; (b) with PE-2 and the rough yeast strain; (c) with PE-2 and *L. fermentum*; and (d) with PE-2, the rough yeast strain and *L. fermentum*.

The fermentation flasks (in duplicate) were maintained at 30 °C for 72 h without shaking and samples of 15 mL were withdrawn at regular intervals of 12 h each, centrifuged at 580*g* for 5 min and then analysed for the presence of total reducing sugars (g 100 mL^−1^), following the methodology of dinitrosalicylic acid by Miller (Miller 1959), after hydrolysis of the sample with hydrochloric acid. Ethanol content (g 100 mL^−1^) following distillation of the samples was determined by measuring the alcohol concentration with a digital densimeter (*Anton*-*Paar*). Ethanol yield (%) was calculated based on the ethanol content of the fermented medium and the consumption of the total reducing sugars in relation to the theoretical efficiency of Gay-Lussac (0.511 g ethanol per gram of glucose). Ethanol productivity (g L^−1^ h^−1^) was determined by computing the ratio of ethanol concentration to the fermentation time. Yield and productivity were calculated based on the mean of the selected time periods (12, 24, 36 and 48 h).

### Fermentative tests in a multiple-cycle batch system

The inoculum was prepared as described above. The fermentations were conducted in 125-mL flasks containing 50 mL of fermentation medium (clarified sugar cane juice or molasses with approximately 14–17 g 100 mL^−1^ of total reducing sugars, pH 4.5—as supplied by a local fuel ethanol-producing unit, and sterilized at 120 °C for 20 min). The flasks were incubated at 30 °C without shaking for six fermentation cycles of 9 h each. All assays were conducted in duplicate. At the end of each cycle, the cell culture was centrifuged and the biomass was collected as inoculum for the next fermentative cycle. The supernatants were analysed as previously described. The chemical analyses were conducted as described previously. Ethanol yield (%) and ethanol productivity (g L^−1^ h^−1^) were calculated based on the mean of fermentative cycles.

### Chromatographic analysis

Concentrations of sucrose, fructose, glucose, acetate, and glycerol in the fermentation samples (collected at the end of the 6th fermentative cycle and filtered with a 0.45 µm porosity membrane) was determined by High Performance Liquid Chromatography (HPLC) comprising of an automatic injector, a refractive index detector, and an exclusion column Varian Hi-Plex H (Agilent Technologies) preceded by a Security Guard Cartridge Carbo-H guard column. The mobile phase consisted of ultra-pure water pumped at a constant flow rate of 0.6 mL min^−1^. Column temperature was maintained at 35 °C and a 20 µL sample volume was injected for the purpose of analysis. The compounds were identified by their relative retention times.

Quantification was achieved by using external standard calibration curves in concentrations of 10, 7.5, 5.0, 2.5, 1.25, 0.625, 0.3125, 0.1562, 0.0781 and 0.0390 g L^−1^ prepared in ultra-pure water. Instrument control and data analysis were conducted using the Empower 3 software. Differences between the calculated and standards concentration values, expressed as a percentage of the standard value (% deviation), varied between negligible and 7.59% for all the compounds that were tested.

### Invertase activity

Invertase activity was determined using whole cells from pure cultures of PE-2 and the rough strain. Cells used for this purpose were cultivated for a 9-h growth period (log phase) in sugarcane juice or molasses containing 4 g 100 mL^−1^ of reducing sugars. These cultures were grown at 30 °C and 160 rpm. Yeast cells were recovered by centrifugation and prepared according to the protocol previously described by Silveira et al. (1996). Glucose concentration was determined using the commercially available Glucose Liquicolor InVitro^®^ kit. The enzyme activity was reported as mmol of glucose per hour per gram of wet biomass. All experiments were carried out in duplicate and at least two independent experiments were performed.

### Kinetics of glucose and fructose consumption

The yeast strains (PE-2 and rough strain) were grown in YPD broth, centrifuged and inoculated in a 500-mL Erlenmeyer flask (in triplicate) containing 200 mL of a sterilized medium consisted of 5 g L^−1^ potassium dihydrogen phosphate, 1 g L^−1^ potassium chloride, 1.5 g L^−1^ ammonium chloride, 1 g L^−1^ magnesium sulphate heptahydrate, 6 g L^−1^ yeast extract, 60 g L^−1^ glucose and 60 g L^−1^ fructose, pH 4.5. The initial cell concentration was approximately 10^7^ CFU mL^−1^. The flasks were inoculated and maintained at 30 °C for 54 h without agitation. Samples of 2 mL were withdrawn each 6 h for the determination of reducing sugar (dinitrosalicylic acid method as cited before) and glucose concentrations (Glucose Liquicolor InVitro^®^ kit). Fructose concentration was obtained by subtracting the glucose concentration from the reducing sugar concentration. The kinetic constant (k, h^−1^) for glucose and fructose consumptions were fitted to a exponential decay function (first-order model), as previously used by Arroyo-López et al. (2008) and Tronchoni et al. (2009), S = S_0_e^−kt^, where S is the content of glucose or fructose (g L^−1^) present in the medium at “t (h)”; and S_0_ is the initial concentration of sugars. The equations obtained were used to calculate the time (h) necessary to consume 50% of the initial sugar concentration present in the medium (t_50_) for glucose and fructose.

## Results

**Fig. 1:**
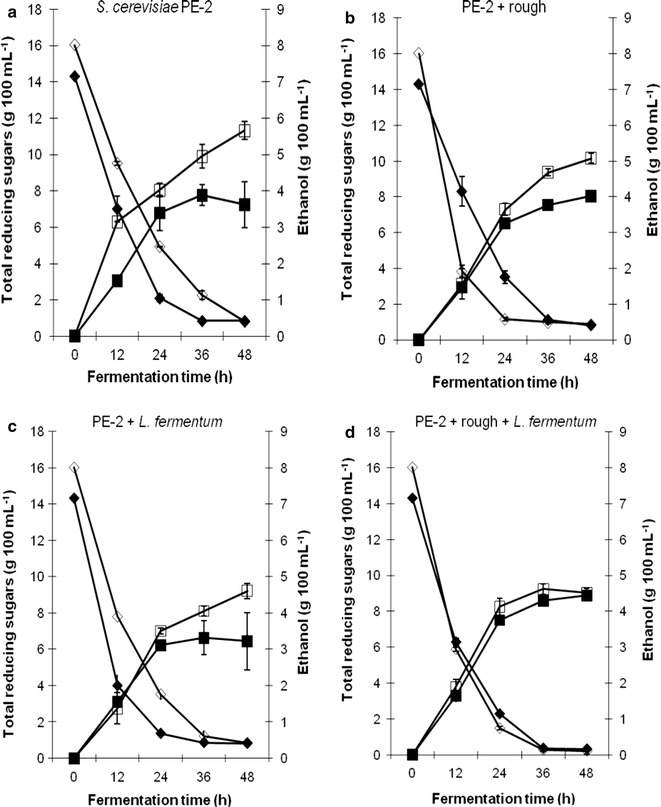
Total reducing sugars (g 100 mL^−1^, diamonds) and ethanol production (g 100 mL^−1^, squares) in fermentations carried out with the industrial *S. cerevisiae* yeast (PE-2) contaminated with the rough strain of *S. cerevisiae* (rough) and/or *L. fermentum* (Lf), in sugarcane juice (empty symbols) or molasses (solid symbols) at 30 °C for 48 h. Results are the mean of two replicates and error bars correspond to the standard deviations

In single-cycle batch fermentations, ethanol production using the pure culture of the industrial PE-2 strain was observed to be higher in sugarcane juice as compared to molasses (Fig. 1a). When the fermentation process was inoculated with either the rough strain of *S. cerevisiae* (Fig. 1b) or with *L. fermentum* (Fig. 1c), the difference in ethanol production between these two substrates was markedly lower. This difference disappeared altogether when both contaminants were introduced in the fermentation process simultaneously (Fig. 1d).

The ethanol production was lower in case of bacterial contamination than with rough yeast contamination (Fig. 1b, c). The double contamination did not result in further lowering of ethanol production values (Fig. 1d) implying that *L. fermentum* did not potentiate the effect of contamination by the rough yeast strain.

In the specific case of sugarcane juice, it was observed that the ethanol yield declined sharply from 77% to approximately 50% when the contaminants were inoculated. This drop in yield was maintained irrespective of the type of contaminant present, associated or not to each other. In case of molasses, the ethanol yield of contaminated and pure fermentations was found to oscillate between 43 and 55%. Ethanol productivity was also seen to decline when contaminants were present in sugarcane juice (1.7–1.2 g L^−1^ h) whereas for molasses productivity was observed to range from 1.1 to 1.3 g L^−1^ h ethanol (Table 1).

**Table 1:** Ethanol yield (%) and ethanol productivity (g L^−1^ h^−1^) of the fermentations carried out with the industrial *S. cerevisiae* yeast (PE-2) contaminated with the rough strain of *S. cerevisiae* (rough) and/or *L. fermentum* (Lf), in sugarcane juice (SCJ) or molasses (MOL) at 30 °C, in single and multiple-batch fermentations

Fermentation	Ethanol yield/productivity	Feedstock	Fermentation trials			
			PE-2	PE-2 + rough	PE-2 + *Lf*	PE-2 + rough + *Lf*
Single-batch fermentation^a^	Yield (%)	SCJ	77.18 ± 11.18	49.86 ± 17.02	50.05 ± 11.75	51.26 ± 9.45
		MOL	51.22 ± 8.47	55.03 ± 4.74	42.95 ± 9.77	55.83 ± 9.92
	Productivity (g L^−1^ h^−1^)	SCJ	1.71 ± 0.60	1.30 ± 0.22	1.17 ± 0.24	1.38 ± 0.33
		MOL	1.13 ± 0.28	1.12 ± 0.21	1.05 ± 0.30	1.26 ± 0.26
Multiple-batch fermentation^b^	Yield (%)	SCJ	87.78 ± 6.82	69.95 ± 22.79	67.67 ± 13.16	82.92 ± 9.57
		MOL	83.30 ± 15.82	67.17 ± 18.94	80.85 ± 11.53	80.98 ± 3.93
	Productivity (g L^−1^ h^−1^)	SCJ	3.22 ± 1.23	2.57 ± 0.78	2.62 ± 1.02	3.18 ± 1.19
		MOL	3.28 ± 1.09	3.03 ± 1.02	3.03 ± 0.97	2.83 ± 0.98

Results are the mean ± standard deviation of eight^a^ or twelve^b^ replicates

These results indicate that the effect of contamination was more prominent in sugarcane juice than in molasses and that the bacterial contamination was more harmful than the rough yeast contamination, when considering single-cycle batch fermentation.

**Fig. 2:**
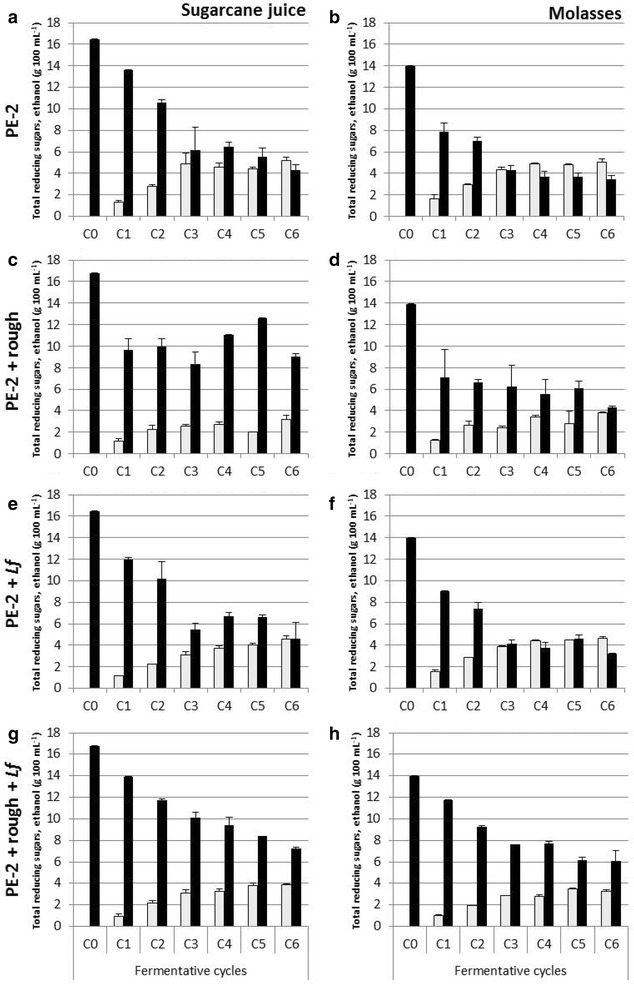
Total reducing sugars (g 100 mL^−1^, black bars) and ethanol production (g 100 mL^−1^, grey bars), in fermentations carried out with the industrial *S. cerevisiae* yeast (PE-2) contaminated with the rough strain of *S. cerevisiae* (rough) and or *L. fermentum* (Lf), in sugarcane juice and molasses, at 30 °C, for six 9-h fermentation cycles. Results are the mean of two replicates and error bars correspond to the standard deviations

In multiple-cycle batch fermentations, the lowest ethanol production and highest residual sugar content were observed in fermentation processes contaminated only with the rough yeast (Fig. 2c, d). When *L. fermentum* was also introduced in the process, no further drop in ethanol production was observed (Fig. 2g), on the contrary, the values were almost always higher than with rough yeast contamination alone. Comparing the feedstocks, in molasses, the residual sugar content was observed to be lower. However, the ethanol production was almost the same when comparing sugarcane juice and molasses for each culture (Fig. 2a–g).

The ethanol yield was observed to be lower in the case of fermentations inoculated with either the rough yeast or with the bacterium (from 88 to 68–70%), except for the fermentation contaminated only with *L. fermentum* in molasses (from 88 to 81%). Interestingly, a higher value, approaching the value observed for the fermentation with no contamination, was seen when both contaminants were present together (81–83%). This effect was observed uniformly for both substrate systems (Table 1).

Ethanol productivity was seen to decrease with contamination but this decrease was similar for both substrates (Table 1). When comparing to numbers obtained in single-cycle batch fermentations, ethanol efficiency and productivity were higher in multiple-cycle batch fermentations (Table 1), which is the usual industrial set-up.

The composition of the fermented broth at the end of the 6th fermentative cycle, which was analysed by HPLC, revealed that for all fermentation trials sucrose was completely hydrolysed. The only exception was observed in fermentations inoculated with *L. fermentum,* wherein 0.22 g 100 mL^−1^ of residual sucrose was detected for both substrates. The amount of residual glucose and fructose was consistently lower for the fermentation without contaminants, irrespective of the feedstocks. As opposed to glucose, a higher concentration of fructose was observed when the residual sugar content of the fermented broth was analysed for all assays in this study (Table 2).

**Table 2:** Composition of the fermented broth at the end of the 6th fermentative cycle in fermentations carried out with the industrial *S. cerevisiae* yeast (PE-2) contaminated with the rough strain of *S. cerevisiae* (rough) and/or *L. fermentum* (Lf) in sugarcane juice or molasses at 30 °C

Composition (g 100 mL^−1^)	Sugarcane juice				Molasses			
	PE-2	PE-2 + rough	PE-2 + *Lf*	PE-2 + rough + *Lf*	PE-2	PE-2 + rough	PE-2 + *Lf*	PE-2 + rough + *Lf*
Sucrose	0	0	0.22 ± 0	0	0	0	0.22 ± 0	0
Glucose (Glu)	1.42 ± 0.36	4.23 ± 0.25	1.76 ± 0.41	2.38 ± 0.07	0.98 ± 0.02	1.66 ± 0.06	1.12 ± 0.03	1.64 ± 0.04
Fructose (Fru)	2.02 ± 0.66	6.11 ± 0.14	2.57 ± 0.77	5.26 ± 0.09	1.56 ± 0.07	3.47 ± 0.06	1.71 ± 0.04	4.47 ± 0.07
Glycerol	0.39 ± 0.02	0.34 ± 0.03	0.40 ± 0.08	0.49 ± 0.02	0.44 ± 0.01	0.45 ± 0.02	0.40 ± 0.04	0.45 ± 0.05
Acetate	0	0.14 ± 0	0.06 ± 0.09	0.20 ± 0	0.21 ± 0	0.26 ± 0.01	0.19 ± 0	0.22 ± 0
Ratio Fru/Glu	1.43	1.44	1.46	2.22	1.59	2.09	1.52	2.73

The analysis was carried out by HPLC

Initial total reducing sugar concentration (in g 100 mL^−1^); 17 (sugarcane juice) and 14 (molasses)

Results are the mean ± standard deviation of two replicates

Changes in the substrate or type of contaminants did not impact glycerol concentration, which was observed to range between 0.34–0.49 g 100 mL^−1^. However, acetate production seemed to vary according to the substrate and type of contaminant and was higher in molasses than in sugarcane juice. Considering each substrate isolatedly, it is noteworthy that the acetate production was always higher when the rough yeast was inoculated into the fermentation (Table 2).

The assays in semi-synthetic medium containing equal amounts of glucose and fructose and inoculated with the strains of *S. cerevisiae* (PE-2 and the rough strain) separately have revealed that both sugars were consumed concomitantly by the yeasts with different rates. The industrial yeast strain consumed glucose and fructose faster than the rough strain. Moreover, the half-time required for glucose and fructose (t_50_) was lower for PE-2 (Table 3).

**Table 3:** Kinetic constants for glucose and fructose consumption (k, in h^−1^) and time to consume half (t_50_, in h) of the initial concentration of glucose and fructose calculated for the *S. cerevisiae* yeasts PE-2 (starter yeast) and the rough strain in semi-synthetic medium containing both glucose and fructose as carbon sources, at 30 °C, without agitation

Culture	Glucose		Fructose	
	k (h^−1^)	t_50_ (h)	k (h^−1^)	t_50_ (h)
PE-2	0.147 ± 0.005	4.71 ± 0.18	0.065 ± 0.001	10.67 ± 0.30
Rough strain	0.090 ± 0.001	7.75 ± 0.91	0.057 ± 0.002	12.10 ± 0.53

Results are the mean ± standard deviation of three replicates

**Fig. 3:**
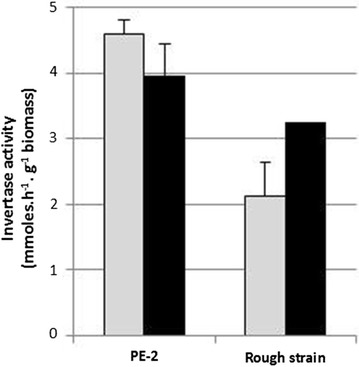
Invertase activity (mmoles glucose hour per gram biomass) displayed by the yeasts PE-2 and the rough strain in sugarcane juice (grey bars) and molasses (black bars) after 9-h growth at 30 °C and 160 rpm with 4 g 100 mL^−1^ of reducing sugars. Results are the mean of two replicates. Error bars correspond to the standard deviations

We also evaluated the invertase activity of both yeast strains in an attempt to verify if differences are found among the yeast strains of *S. cerevisiae* and or between the substrates. Invertase activity was determined in whole yeast cells obtained from pure cultures of both yeasts using sugarcane juice and molasses as the growth medium. Invertase activity was observed to be lower in the rough strain culture as compared to the PE-2 strain. This result was observed unanimously regardless of the substrate used. However, the degree of difference varied depending upon the substrate used. In case of PE-2, the invertase enzyme activity was 14% higher in sugarcane juice comparing to molasses. Conversely, the invertase activity was 60% higher when molasses was used as the substrate by the rough yeast strain (Fig. 3).

## Discussion

The results here indicate that in multiple-cycle batch system, the feedstock had a minor impact on the fermentation than in single-cycle batch system, however the rough yeast contamination was more harmful than the bacterial contamination in multiple-cycle batch fermentation. It occurred regardless the feedstock utilized. This result may be explained by the fact that the rough yeast presents slow fermentation (Reis et al. 2013) and the duration of the cycles was 9 h, similar to industrial conditions (Basso et al. 2008). The inoculation of both contaminants simultaneously had no higher effect on the fermentation than the contaminant inoculated one at a time, in both fermentation systems.

The fermentation of sugarcane molasses might display some particular features (Pereira et al. 2014). Acetate production by *D. bruxellensis* was observed in molasses but not in sugarcane juice, as we also verified here, under conditions similar to the ones employed in this study. The presence of some unknown final electron acceptor may account for this metabolite to be produced only in the case of molasses but not in sugarcane juice medium, as there are clear differences in the chemical composition of both substrates (Pereira et al. 2012, 2014). We observed only punctual differences between the feedstocks regarding the contaminants.

In the fermentative processes a multitude of interactions between bacterial and yeast cells is very frequently observed. Bacteria can induce flocculation of yeast cells and may selectively promote the growth of a non-*Saccharomyces* yeast rather than a *Saccharomyces* strain. In case of *D. bruxellensis* and *Lactobacillus vini*, these interactions exert their effect in a fashion that benefits the contaminant yeast over the starter yeast. This variation was attributed to differences in the mannose content of yeast cell wall (Tiukova et al. 2014). It is well known that contamination of ethanolic fermentations by *L. fermentum* (Basso et al. 2008; Carvalho-Netto et al. 2015) results in cellular co-aggregation, high production of organic acids and decrease in ethanol yield, but these studies were carried out in fermentations without any other contaminant but *L. fermentum.* Yeast cell flocculation was not observed in the fermentations evaluated in this study but decrease in ethanol production was. Our results suggest that an interaction between the rough colony contaminant strain of *S. cerevisiae* and *L. fermentum* should have occurred in the conditions here studied.

Higher residual sugar concentration, lower ethanol productivity and yield are characteristics commonly associated with the presence of rough yeast in the fermentation medium in multiple-cycle fermentation (Basso et al. 2008; Reis et al. 2013), resulting in slow fermentation rate. We then speculated the causes behind the sluggish fermentation rate displayed by the rough yeast focusing in the sugar consumption rate and invertase activity.

It is well established in literature that glucose is the substrate of choice for *S. cerevisiae* (Berthels et al. 2004) and consumed first. Consequently, residual sugar in the fermented broth usually contains more fructose than glucose. The same was also observed in this study. However, it was noticed that in fermentations that were contaminated with the rough yeast, the residual fructose concentration was much higher, with higher discrepancy between glucose and fructose concentrations (ratio fructose to glucose), especially in molasses. From the results, it could be inferred that the fructose consumption by the rough yeast is slower than the fructose consumption by the industrial yeast PE-2. Our experiments in semi-synthetic medium with glucose and fructose demonstrated indeed that both fructose and glucose consumption rates are lower for the rough yeast strain compared to the industrial strain of *S. cerevisiae*.

Stuck fermentation in the wine industry is commonly associated with high fructose/glucose ratio (Gafner and Schutz 1996; Tronchoni et al. 2009). Changes in the kinetics of sugar consumption were observed during the increase of ethanol content in the medium, which increased the glucose metabolism rate higher than that observed for fructose (Zinnai et al. 2013). The evolution of ethanol production along the fermentation time may affect the rates in which glucose and fructose are consumed and it seemed that this effect is different on the industrial yeast strain comparing to the rough yeast contaminant.

Concerning invertase, this enzyme is encoded by the *SUC2* gene. Two variants of the enzyme are produced: (a) a glycosylated form (regulated by glucose repression) that is secreted into the periplasmic space, and (b) a non-glycosylated variant that is constitutively produced and present within the cytosol. The glycosylated form represents most of the observed enzyme activity in the de-repressed cells (Carlson and Botstein 1982). It is worth considering that lower invertase activity could also account for the slower fermentation rate in fermentations that are contaminated with the rough yeast strain. However, the fact that all the sucrose was hydrolysed within 9 h of fermentation can probably be credited to the presence of PE-2 in the co-culture. So, the observed difference in invertase activity by the rough yeast strain may not be the cause behind the slow fermentation rate displayed in fermentations contaminated by this yeast strain.

The values of glycerol production in this study were similar to those reported by Basso et al. (2014), wherein *L. fermentum* was cultivated along with the industrial yeast strain CAT–1 in a mixed substrate, compounded by sugarcane juice and molasses for five fermentative cycles (0.4–0.6 g 100 mL^−1^). Regarding acetate production, many factors such as yeast strain, sugar concentration, fermentation temperature, pH and aeration, can affect the acetate production in wine fermentation (Chidi et al. 2015), but in ethanolic fermentation for fuel ethanol production, these factors have not been studied so far.

The production of acetic acid is correlated with the production of glycerol in the wine industry. The shift in redox balance (NADH:NAD^+^ ratio) caused by higher glycerol production (in response to changes such as alteration in osmotic pressure for example) is corrected through acetic acid production as a redox sink to convert NAD^+^ back to NADH (Remize et al. 1999; Erasmus et al. 2004). However, the acetic acid production was more dependent on the yeast strain (Erasmus et al. 2004; Chidi et al. 2015). In this study, we were unable to find a positive correlation between glycerol and acetate production.

Based on the results obtained in this study, it appears that the major issues associated with incomplete and slow fermentation in contaminated fermentations are related to the slower sugar consumption rates, acetate production and also possibly to the diminished preference for fructose by the rough yeast strain comparing to the industrial yeast strain. These factors may be instrumental in impairing fermentation when this particular yeast strain is present in the medium. Whether it is the cellular morphology displayed by the rough yeast strain that compromises the sugar uptake or the physiological differences concerning hexose transporters, the real reasons behind the fermentation characteristics of sluggish fermentation remain unclear and must be further investigated.

The role of the bacterium *L. fermentum*, in a scenario in which fermentation was carried out by an industrial strain like PE-2 whilst being contaminated by a rough yeast strain, was not to potentiate the harmful effects of the contamination by the wild yeast strain regardless of the feedstock. There is no doubt that this result is very interesting in the context of Brazilian industrial fermentation. However, even considering that the ethanol yield was re-established with the inoculation of *L. fermentum*, there are numbers that should not be despised: decreases of approximately 5.7% in ethanol yield and 1.2% in ethanol productivity, and increase in 122% in the residual sugar content in sugarcane juice, in the fermentation contaminated with both rough yeast and *L. fermentum* compared to the fermentation without contamination; for molasses, the numbers are 2.4, 13.7 and 140%, respectively. Strategies to control or eliminate the contaminants are highly demanded and they are not yet available in case of the rough yeast contamination, unfortunately. For bacterial contamination, the acid treatment of the cells between the fermentative cycles is able to decrease the number of viable cells and an alternative treatment based on the addition of ethanol to the acid solution has been successfully proposed (Costa et al. 2017).
